# Alterations in HLA Class I-Presented Immunopeptidome and Class I-Interactome upon Osimertinib Resistance in EGFR Mutant Lung Adenocarcinoma

**DOI:** 10.3390/cancers13194977

**Published:** 2021-10-04

**Authors:** Yue A. Qi, Tapan K. Maity, Shaojian Gao, Tao Gong, Meriam Bahta, Abhilash Venugopalan, Xu Zhang, Udayan Guha

**Affiliations:** 1 Thoracic and GI Malignancies Branch, Center for Cancer Research, NCI, NIH, Bethesda, MD 20892, USA; tapan.maity@nih.gov (T.K.M.); james.gao@nih.gov (S.G.); johny4432318@126.com (T.G.); meriam.bahta@nih.gov (M.B.); abhilash.venugopalan@nih.gov (A.V.); xu.zhang@nih.gov (X.Z.); 2 Center for Alzheimer’s and Related Dementias, National Institute on Aging, NIH, Bethesda, MD 20892, USA; 3 Bristol-Myers Squibb, Lawrenceville, NJ 08901, USA

**Keywords:** HLA, immunopeptidome, antigen presentation, SILAC, proteomics, immune evasion, osimertinib resistance, lung adenocarcinoma

## Abstract

**Simple Summary:**

We sought to identify molecular mechanisms of lower efficacy of immunotherapy in epidermal growth factor receptor (EGFR) mutant lung adenocarcinoma and the differences in those mechanisms with the emergence of tyrosine kinase inhibitor (TKI)-resistance. To this end, we conducted affinity purification and quantitative mass spectrometry-based proteomic profiling of human leukocyte antigen (HLA) Class I-presented immunopeptides and Class I-interacting proteins. This large-scale dataset revealed that the Class I-presented immunopeptidome was suppressed in two third-generation EGFR TKI, osimertinib-resistant lung adenocarcinoma cell lines compared to their isogenic TKI-sensitive counterparts. The whole-cell proteomic profiling show that antigen presentation complex proteins and immunoproteasome were downregulated upon EGFR TKI resistance. Furthermore, HLA class I-interactome profiling demonstrated altered interaction with key apoptosis and autophagy pathway proteins. In summary, our comprehensive multi-proteomic characterization in antigen presentation machinery provides potentially novel evidence of poor immune response in osimertinib-resistant lung adenocarcinoma.

**Abstract:**

Immune checkpoint inhibitor (ICI) therapy has been a paradigm shift in the treatment of cancer. ICI therapy results in durable responses and survival benefit for a large number of tumor types. Osimertinib, a third-generation epidermal growth factor receptor (EGFR) tyrosine kinase inhibitor (TKI) has shown great efficacy treating EGFR mutant lung cancers; however, all patients eventually develop resistance. ICI therapy has not benefitted EGFR mutant lung cancer. Herein, we employed stable isotope labeling by amino acids in cell culture (SILAC) quantitative mass spectrometry-based proteomics to investigate potential immune escape molecular mechanisms in osimertinib resistant EGFR mutant lung adenocarcinoma by interrogating the alterations in the human leukocyte antigen (HLA) Class I-presented immunopeptidome, Class I-interactome, and the whole cell proteome between isogenic osimertinib-sensitive and -resistant human lung adenocarcinoma cells. Our study demonstrates an overall reduction in HLA class I-presented immunopeptidome and downregulation of antigen presentation core complex (e.g., TAP1 and ERAP1/2) and immunoproteasome in osimertinib resistant lung adenocarcinoma cells. Several key components in autophagy pathway are differentially altered. S100 proteins and SLC3A2 may play critical roles in reduced antigen presentation. Our dataset also includes ~1000 novel HLA class I interaction partners and hundreds of Class I-presented immunopeptides in EGFR mutant lung adenocarcinoma. This large-scale unbiased proteomics study provides novel insights and potential mechanisms of immune evasion of EGFR mutant lung adenocarcinoma.

## 1. Introduction

Cancer immunotherapy has achieved less success in EGFR mutant lung cancers [1,2]. Osimertinib, a third generation EGFR TKI, has shown great efficacy in EGFR mutant lung adenocarcinoma; however, patients treated with osimertinib eventually develop acquired resistance [3,4]. ICI therapy has been ineffective as second line therapy in EGFR mutant lung adenocarcinoma ([5]). The combination of immune checkpoint inhibitors (ICI) and EGFR TKIs have undergone several investigations and clinical trials without much added benefit, while having significant immune-related adverse events (irAE) [6,7]. Clinical studies showed that combination of osimertinib and durvalumab, an anti-programmed death ligand 1 (PD-L1) antibody, did not significantly benefit the patients compared to osimertinib alone while further increasing pneumonitis and other irAEs [8]. Emerging evidence suggests that TKIs may cause immunosuppression and in some contexts even reduce PD-L1 expression in EGFR mutant lung tumors. However, the molecular mechanism of immune escape has not been elucidated [9,10,11]. To this end, and to interrogate potential alterations in antigen processing and presentation, we used quantitative mass spectrometry (MS)-based proteomic analysis to globally profile the landscape of human leucocyte antigen (HLA) Class I-presented immunopeptidome, the total proteome, and the Class I-interactome in EGFR-mutant lung adenocarcinoma cell lines and isogenic osimertinib-resistant (OsiR) counterparts.

MS-based peptide sequencing has been widely used for high throughput MHC-associated peptidome discovery [12,13,14,15]. To systematically and accurately quantify the HLA associated immunopeptides presented on the tumor cell surface, we leveraged stable isotope labeling by amino acids in cell culture (SILAC) and mass spectrometry (MS)-based proteomics. This approach has been employed to quantitively profile HLA peptidome to study the impact of proteasomal inhibition in antigen presentation [16,17]. Our group uncovered novel therapeutic biomarkers using SILAC-based quantitative proteomics [18,19]. Here, the metabolically labeled immunopeptides in steady-state from osimertinib-sensitive and resistant lung adenocarcinoma cells were enriched and analyzed using pan-HLA class I antibody-based affinity purification-mass spectrometry (AP-MS). We employed a novel method using low percentage organic buffer for Class I-presented peptide elution and high percentage organic buffer for the elution of proteins in the HLA protein complex—the direct and indirect interaction partners of HLA Class I. We also quantified the global proteome to characterize the potential contribution of protein expression differences in antigen processing and presentation. Overall, we designed a comprehensive SILAC-based quantitative multi-proteomic workflow for large-scale profiling of the whole-cell proteome, HLA class I-immunopeptidome, and the HLA Class I-interactome in a single experiment. We employed multiple informatic tools to visualize and discover the altered antigen processing and presentation pathway, integrity of proteasome assembly, autophagy, and protein degradation pathways.

## 2. Materials and Methods

### 2.1. Cell Culture and SILAC Labeling

EGFR mutant lung adenocarcinoma cell lines, PC9 and H1975, were obtained from ATCC and Varmus Laboratory (MSKCC), respectively; their OsiR counterparts, PC9-OsiR and H1975-OsiR, were generated in-house after long-term and step-wise increase in osimertinib exposure (Figure S1a). As described previously [20], PC9 and H1975 cells were plated at low density (0.5 million/10 cm dish) and were treated with 25 nM osimertinib for 2 days that resulted in cell death of the majority of the cells. The surviving cells were subsequently grown in increasing concentrations of osimertinib (up to 2.0 μM) for 2 weeks. The newly generated osimertinib resistant PC9-OsiR and H1975-OisR cells were maintained in complete cell growth medium supplemented with 2.0 μM osimertinib. The SILAC labeling protocol was described by our group previously [18]. Briefly, the base cell growth medium was SILAC-RPMI 1640 medium (Pierce, Rockford, IL, USA) supplemented with 10% dialyzed fetal bovine serum (Invitrogen, Carlsbad, CA, USA); for PC9 and H1975 cell lines, light isotopic labeled L-lysine and L-arginine (Sigma-Aldrich, St. Louis, MO, USA) were added to the base cell culture medium; for PC9-OsiR and H1975-OsiR cells, heavy isotopic labeled ^13^C_6_^15^N_2_-Lysine (Lys8) and ^13^C_6_^15^N_4_-Arginine (Arg10) (Cambridge Isotope laboratories, Tewksbury, MA, USA) were supplemented to the base medium. The cells were initially cultured in corresponding SILAC medium for at least 6-doublings and were subjected to incorporation rate checking. After complete labeling (i.e., >95%), we kept the cells in SILAC cell growth medium for the following subculture.

### 2.2. Cell Viability Assay

The lung adenocarcinoma cell lines, PC9 and H1975, and their osimertinib resistant counterparts, PC9-OsiR and H1975-OsiR, were seeded in a 96-well tissue culture plate at 4000 cells/well. The cells were treated with serial concentrations of osimertinib for 72 h, which were 0.001, 0.1, 1.0, 10, 50, 100, 1000, 10,000 nM of osimertinib. This experiment included 3 biological replicates per concentration per cell line. Subsequently, we added 50 μL of Promega CellTiter-Glo^®^ Luminescent Cell Viability Assay reagent, followed by 15 min incubation at room temperature. The raw luminescence was acquired by SoftMax Pro 5.4.1 on a microplate reader, and then was normalized and plotted with GraphPad Prism.

### 2.3. Affinity Purification of HLA Class I Immunopeptidome and Interactome

We collected 2.0 × 10^8^ cells per cell line per replicate (*n* = 3) in ice-cold lysis buffer (20 mM Tris-HCl pH = 8.5, 100 mM NaCl, 1 mM EDTA. 1% triton X-100 supplemented with Halt 1:100 protease Inhibitor cocktail Cat. No 78430, Thermo Scientific). After 30 min incubation and rotation at 4 °C, the cell lysate was centrifuged at 20,000× *g* for 2 h at 4 °C. The supernatant was collected and subjected to bicinchoninic acid assay (BCA) for protein concentration evaluation. Equal amounts of light and heavy labeled cell lines (i.e., PC9-OsiR and PC9 or H1975-OsiR and H1975) were mixed, and 90% of the final protein mix was subject to following HLA class I affinity purification, the remaining 10% was used for whole-cell proteomic profiling. From here, we had both OsiR and their parental lines processed in single tubes for the downstream sample preparation. Prior to the HLA enrichment, 0.5 mg HLA Class | pan antibody clone W6/32 (BioXcell, West Lebanon, NH) were incubated with 200 μL slurry of protein A/G agarose beads (Santa Cruz Biotechnology, Dallas, TX, USA) for 12 h at 4 °C. Then, we added the W6/32 pre-coupled resin to the mixed SILAC samples and incubated for 12 h at 4 °C. To reduce non-specific binding and background, stringent washes to the beads were conducted 6 times using ice-cold wash buffers including 3 washes with lysis buffer, 2 washes with 20 mM Tris-HCl (pH = 8.5) and 1 wash with pure water. The HLA class I-peptide complexes were eluted 4 times using mild acid 0.15% trifluoroacetic acid (TFA) in water at room temperature, following by purification of immunopeptides and class I interaction proteins. For the immunopeptides, combined mild acid eluates were loaded to preconditioned C_18_ desalting resin columns following by twice washing with 0.1% TFA in water, and twice elution with 30% acetonitrile (ACN) in 0.1% TFA, combining eluates were subjected to speed vacuum drying. For the interactome enrichment, twice elution with 80% ACN in 0.1% TFA were collected right after the low% ACN elution, following by pre-frozen and overnight lyophilization at −80 °C. The lyophilized peptides were reconstituted in 2% ACN in 0.1% TFA (MS sample loading buffer) and were subject to LC-MS/MS analysis. The lyophilized proteins were stored in −80 °C. We innovated this two-step elution to enrich both HLA binding peptides and interaction proteins. Typical peptides are less hydrophobic than proteins, so peptides are easier to be eluted off from reverse phase resin (e.g., C_18_) by low organic buffer.

### 2.4. Tryptic Digestion and Desalting for Total Proteome and HLA Interactome

To increase the protein solubility, the 10% of total mixed cell lysate was mixed 9 M urea Tris-HCl (pH = 8.5) buffer to make 6 M urea cell lysate as the final concentration. For protein reduction and alkylation, we used 10 mM tris(2-carboxyethyl) phosphine hydrochloride (TCEP) with 30 min incubation at 37 °C, following by 20 mM iodoacetamide (Sigma-Aldrich, St Louis, MO, USA) with 30 min incubation in dark at room temperature. We further diluted the samples to 2 M urea with 20 mM Tris-HCl (pH = 8.5) buffer. We added trypsin/lysC (Promega, Madison, WI, USA) per replicate at 1: 50 ratio to total protein followed by 16-h incubation with 1200 rpm at 37 °C on a Thermomixer. For the HLA interaction proteins from previous step, we reconstituted the lyophilized proteins in 2 M urea Tris-HCl (pH = 8.5) buffer. Similar reduction and alkylation steps were performed, following by 1 μg trypsin/lysC digestion per replicate. The tryptic peptides were acidified to 1% TFA and were subjected to C_18_ desalting. Briefly, C_18_ resin column (Waters, Milford, MA, USA) was primed and conditioned by 100% ACN and 0.1% TFA in water, respectively. Then, we loaded the tryptic peptides and washed the column twice with 0.1% TFA in water. The final cleanup peptides were eluted off by 70% ACN in 0.1% TFA and were speed vacuum dried. The peptides derived from total proteome were subjected to offline fractionation, and the peptides derived from HLA interactome were resuspended in MS sample loading buffer for MS analysis.

### 2.5. Offline High-pH Reverse Phase Fractionation and Consolidation of Peptide Fractions

To enhance the proteome coverage, we employed offline peptide fractionation. High-pH reverse phase fractionation was conducted using an XBridge C_18_, 250 × 4.6 mm^2^ analytical column containing 5 μm particles (Waters, Milford, MA) with a flow rate of 1 mL/min. The 48 min separation gradient was from 10% to 45% phase B (100% ACN in 10 mM triethylammonium bicarbonate, TEAB), 10 mM TEAB as phase A. A total of 96 fractions (0.5 min/fraction) were collected in a deep 96 well plate and were concatenated to 12 fractions by 1, 13, 25, 37, 49, 61, 73, 85; and so on. The 12 pooled fractions were lyophilized at −80 °C, and dried peptides were reconstituted in MS sample buffer for MS analysis.

### 2.6. Liquid Chromatography-Tandem MS (LC-MS/MS) Analysis

For the total proteome and interactome, tryptic peptides were first loaded to a 2 cm trap column and were separated by a 250 mm × 75 μm nano analytical C_18_ column for 90 min effective gradient with 4–35% ACN in 0.1% formic acid on an Ultimate 3000 NanoLC (Thermo Scientific, Waltham, MA, USA). Second, tandem mass spectrometer (Orbitrap Q-Exactive HF, Thermo Fisher Scientific) was set MS1 scan at 120,000 resolution, and MS/MS scan at 30,000 resolution with maximum injection time 35 ms. The top 20 most abundant precursors with 2–6 charges were subjected to MS/MS fragmentation using high collision dissociation (HCD) with 35% energy and dynamic exclusion was set to 30 s. For the immunopeptidome, LC setting was the same as total proteome-derived peptides. Due to relatively low abundance of the HLA binding peptides, we used MS/MS scan at 60,000 FWHM resolution with maximum injection time 60 ms. The top 15 most abundant parental ions with 1–4 charges were subjected to MS2 scan, and dynamic exclusion was set to 20 s.

### 2.7. Database Search Analysis

The MS raw files were searched against Uniprot Human proteome reference database (v20170207) that contains 70,948 entries including isoforms using PEAKS studio (v8.5) [21]. The mass tolerance for precursor ions was set to 4.5 ppm, and mass tolerance for fragment ions was set to 20 ppm. For SILAC quantitation module, lys8/arg10 and auto normalization (total peptide normalization of light and heavy peptides) were chosen. For the neutral HLA peptides, no enzyme was selected as digestion enzyme; for the total proteome and interactome, trypsin and LysC were selected as digestion enzyme. False discover rate (FDR) of protein and peptide level identification was chosen at 0.01. All offline fractions were combined into one file for the data output.

### 2.8. Bioinformatic and Statistical Analysis

For the HLA typing, we used Seq2HLA package [22] to identify the four-digit HLA Class I alleles from the whole exome sequencing (WES) of all samples. To remove the common AP-MS nonspecific binding proteins, we leveraged the CRAPome database by filtering out a list of background contamination from normal IgG and agarose beads [23]. The motif analysis of immunopeptidome was conducted by iceLogo [24]. The HLA epitope binding predication was performed using NetMHCpan4.0 [25] and IEDB database [26]. The quantitative SILAC proteomic data were analyzed by MS data post-analysis informatic package, Perseus, including one-sample *t*-test *p*-value and volcano plots visualization [27]. The proteins/peptides, detected in only one SILAC state or cell line group (either in OisR or parental lines) were given a capped ratio H/L 256 for the ones identified only in OsiR (heavy labeled), and 0.01 for the ones identified only in OsiS (light labeled). For the pathway analysis, we leveraged Ingenuity Pathway Analysis (IPA), David bioinformatics package and STRING using multiple databases including GO, KEGG and reactome [28,29,30,31,32]. The interactome network was constructed by Cytoscape (v3.8.1) embedded with ClueGo (v2.5.7) and CluePedia (v1.5.7) [33].

## 3. Results

### 3.1. Identification and Quantification of SILAC Labeled HLA Class I-Associated Peptides

**Figure 1:**
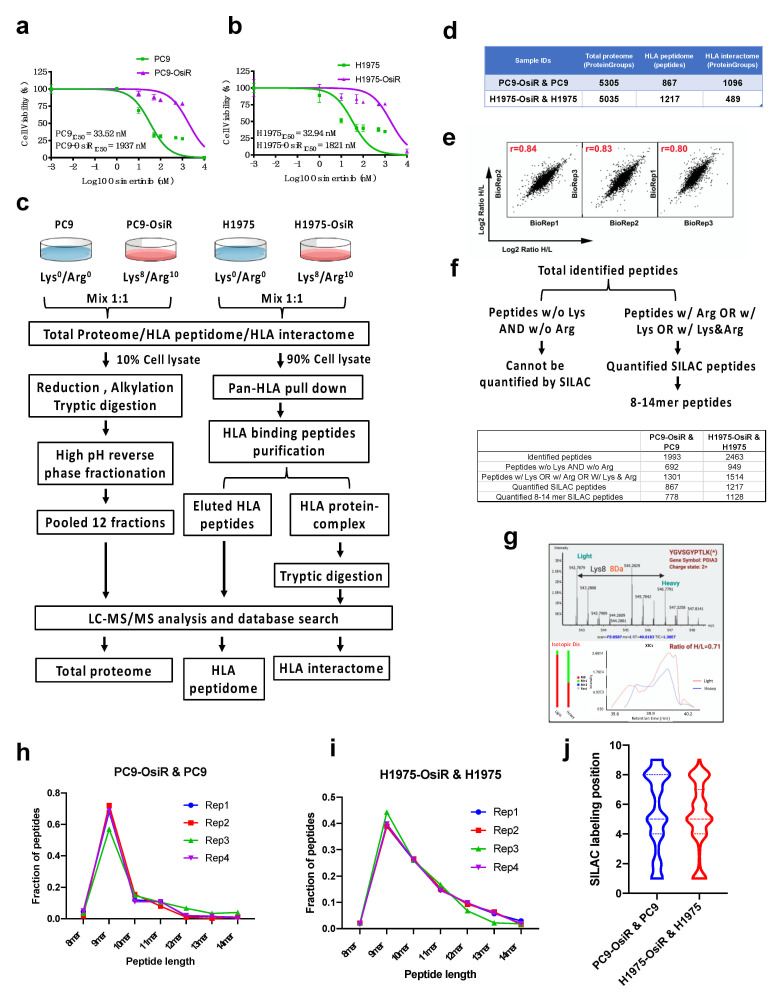
SILAC-based quantitative proteomic profiling of HLA class I-associated peptides. (**a**,**b**) Cell viability assay of PC9 and PC9-OisR (**a**) and H1975 and H1975-OsiR (**b**) under a serial concentration of osimertinib (0.001~10,000 nM). (**c**) Schematic workflow of SILAC-based quantitative proteomic analysis of total proteome, HLA immunopeptidome and HLA interactome in PC9-OsiR/PC9 and H1975-OsiR/H1975 EGFR mutant lung adenocarcinoma cells. (**d**) Summary table of total identifications of whole-cell proteome, HLA peptidome and HLA interactome. (**e**) Representative example of correlation analysis among biological replicates; correlation analysis of total proteome of PC9-OsiR/PC9 is shown. (**f**) Schematic of discovery pipeline of SILAC quantified HLA class I-presented peptides (upper panel) and summary table of the number of peptides after each step of filtering (lower panel). (**g**) Upper panel: an example of SILAC labeled HLA immunopeptide, PDIA3-derived peptide, YGVSGYPTLK (*) with a heavy labeled lysine (lys8), displays the light (osimertinib-sensitive) and heavy (osimertinib-resistant) peaks in a MS full scan, where monoisotopic peak (2+ charged) shows 4Da difference m/z ratio (8Da divided by charge. Lower panel: extracted ion chromatography (XICs) of both light and heavy peptides of YGVSGYPTLK shows a ratio H/L = 0.71, indicating this peptide abundance is reduced by 1.4-fold in the OsiR cells. (**h**,**i**) Peptide length distribution of the identified HLA immunopeptides in 4 biological replicates in PC9-OsiR/PC9 cells (**h**) and H1975-OsiR/H1975 cells (**i**). (**j**) SILAC labeling position (lys and/or arg) distribution in the identified 9 mer peptides in both cell lines.

We hypothesized that there are inherent differences in immunopeptide processing and presentation between EGFR TKI-sensitive and -resistant EGFR mutant lung cancer. The overall goal of this study was to characterize the alterations in antigen processing and presentation upon EGFR TKI resistance. To create cell line models of lung cancer patients with acquired resistance to osimertinib, we developed osimertinib resistance in the EGFR mutant osimertinib-sensitive lung adenocarcinoma cell lines, PC9, harboring the EGFR^Del E746–A750^ mutant, and H1975, harboring the EGFR^L858R/T790M^ mutant by long-term culture of sensitive cells in the presence of increasing concentrations of osimertinib [20]. The isogenic osimertinib-resistant counterparts were PC9-OsiR and H1975-OsiR, with osimertinib IC_50_ values of 1.9 μM and 1.8 μM, respectively (Figure 1a,b). To investigate potentially altered HLA class I immunopeptidome upon osimertinib resistance, we developed a SILAC-based quantitative proteomic analysis workflow (Figure 1c). We aimed to observe the altered immunopeptidome by native peptidome profiling, and further study the molecular mechanisms using total proteome and HLA interactome characterizations. The parental cell lines, PC9 and H1975, and the osimertinib-resistant counterparts, PC9-OsiR and H1975-OsiR, were metabolically labeled with light-and heavy-lysine/arginine, respectively, until confirmed to be labeled to >98%. A small portion (~10%) of the harvested cell lysate was digested with trypsin/lysC and fractionated for total MS proteome profiling. The majority of the cell lysate was subjected for pan-HLA class I immunoprecipitation, and the enriched HLA class I-presented peptides were further eluted by mild acid treatment. The immunoprecipitated HLA protein complexes were further digested with trypsin to quantify the HLA Class I proteins and their interaction partners. More than 5000 protein groups were identified in both cell lines (Table S1). There were 867 and 1217 unique HLA Class I-presented SILAC-labeled peptides quantified in PC9/PC9-OsiR and H1975/H1975-OsiR cells, respectively (Table S2). We also identified 1515 and 711 HLA class I-interacting proteins in these two cell lines, respectively (Figure 1d and Table S3). The reproducibility among biological replicates (*n* = 3) was evaluated by log2 normalized SILAC ratio H/L; the Pearson’s correlation coefficient of PC9 total proteome samples was >0.8 (Figure 1e). Given the fact that not all endogenous immunopeptides contain lysine and/or arginine, we identified 1301 (65%) out of total 1993 identified peptides and 1514 (61%) out of 2463 identified peptides containing at least one lysine or arginine in PC9/PC9-OsiR cells and H1975/H1975-OsiR cells, respectively. Of these, 867 and 1217 peptides were quantified using the SILAC approach having a valid SILAC ratio from the PC9/PC9-OsiR and H1975/H1975-OsiR experiments, respectively. More importantly, among the SILAC quantified Class I-presented peptides, 778 (90%) and 1128 (93%) peptides from PC9/PC9-OsiR and H1975/H1975-OsiR cells contained between 8 to 14 amino acid residues (i.e., 8–14 mer) (Figure 1f). The co-eluted light and heavy labeled peptides were quantified based on their MS1 spectra of precursor ions. For example, protein disulfide-isomerase A3 (PDIA3)-derived peptide YGVSGYPTLK was labeled on the lysine which resulted in a heave peptide with 8 Da molecular weight difference in the OsiR cells. The MS/MS spectra identified the light and heavy labeled precursor ion peaks and confirmed reduction of intensity of the heavy peptide (Figure 1g). We confirmed that 9 mer peptide with 9 amino acids was the most frequent peptide length as reported previously using label free quantitation for Class I presentation [13]. High reproducibility was observed among independent biological replicates in both cell lines (Figure 1h,i). The SILAC labeled positions on Arg or Lys in 9 mer peptides least frequently occurred on known HLA class I peptide anchor positions 2 and 9 (Figure 1j).

### 3.2. HLA Class I Alleles and the Binding Characteristics of the HLA Class I-Presented Immunopeptidome

**Figure 2:**
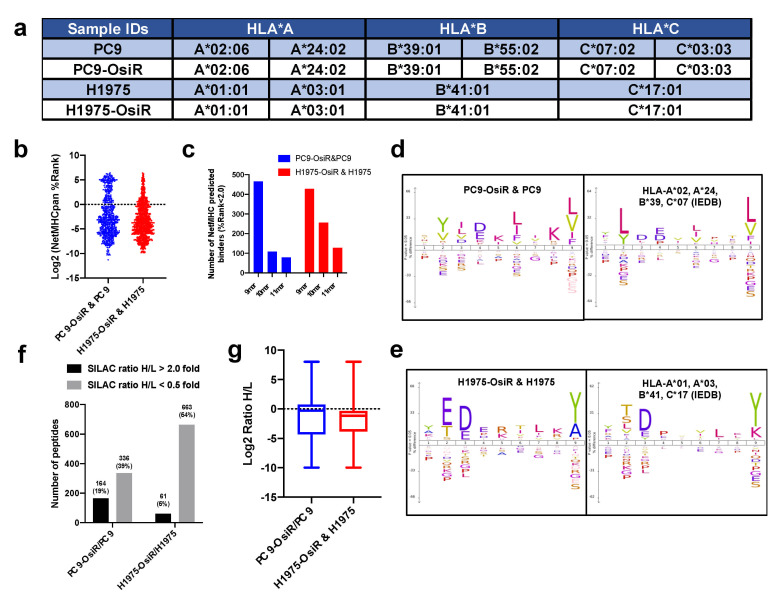
Characterization and quantification of SILAC–labeled HLA immunopeptides. (**a**) Summary table of HLA typing analyzed from whole exome sequencing (WES) data using Seq2HLA. (**b**) Distribution of predicted binding (%rank) of identified immunopeptides using NetMHCpan4.0. (**c**) Number of NetMHCpan predicted binders among identified 9–11 mer peptides in both cell lines. (**d**,**e**) Motif analysis of 9 mer peptides in our study (left panel) and that of corresponding monoallelic binding epitopes reported in IEDB database (right panel). For example, HLA typed alleles of PC9-OsiR/PC9 cells, HLA-A*02, A*24, B*39 and C*07, were used to retrieve their binding peptides in IEDB, and motif analysis of every single allele was overlaid in the right panel of (**d**). Similar analysis was conducted for the H1975-OsiR/H1975 experiment (**e**). (**f**) Overall number of peptides with increased or reduced presentation in OsiR cells compared to sensitive cells (cutoff = 2.0 and 0.5, respectively). (**g**) Box plots show the distribution and median values of log2 SILAC H/L ratios of Class I-presented peptides in PC9-OsiR/PC9 and H1975-OsiR/H1975 lung adenocarcinoma cells.

To leverage computational T-cell epitope prediction algorithms for further characterization, HLA serotyping was performed. We found no change in HLA typing between the osimertinib-sensitive and -resistant isogenic cells. Loss of heterozygosity (LOH) of *HLA-A* and *HLA-B* alleles was observed in H1975 and H1975-OsiR cells (Figure 2a). The NetMHCApan-4.0 [25] prediction algorithm was used to predict binding affinity (i.e., %Rank, lower the rank, higher the binding affinity) of the identified immunopeptides against the serotyped HLA alleles in the respective cell lines. A majority of the 9–11 mer peptides showed that their binding affinity was below the strong binder cutoff (%Rank = 2.0), and 9 mer peptides comprised of the highest number of predicted strong binders (Figure 2b,c, Table S4). When we applied a motif analysis algorithm to the identified 9 mer peptides in our samples and compared with the previously reported 9 mer peptides bound to the HLA-alleles in respective cell lines in the Immune Epitope Database (IEDB) (iedb.org), we found great similarity between these binding motifs (Figure 2d,e). When comparing the multi-allelic motif with their corresponding mono-allelic motifs, the results suggest HLA-A and -B may contribute more to their overall binding motifs than HLA-C (Figure S1b–e). In summary, we identified the Class I-presented immunopeptidome by mass spectrometry and a major fraction of these peptides, quantified by the SILAC approach, showed the properties of HLA class I binders.

Next, we quantified the SILAC-labeled peptidome using normalized heavy/light ratios (i.e., OsiR/parental cells) with a stringent 2.0- or 0.5-fold change cutoff for increased or decreased peptide presentation, respectively. The peptide presentation of 326 peptides (39% of total peptides) was reduced while 164 peptides (19%) was increased in PC9-OsiR compared to PC9 cells. Similarly, 663 peptides (54%) showed reduced peptide presentation while only 61 peptides (5%) had increased presentation in H1975-OsiR cells compared to the H1975 parental cells (Figure 2f). The overall abundance intensity distribution of the Class I presented immunopeptides showed that the median values of log2 Ratio H/L for both cell lines were below zero, indicating that the overall Class I peptide presentation was reduced in OsiR lung adenocarcinoma (Figure 2g).

### 3.3. Correlation of Class I-Presented Peptides and Their Source Proteins

**Figure 3:**
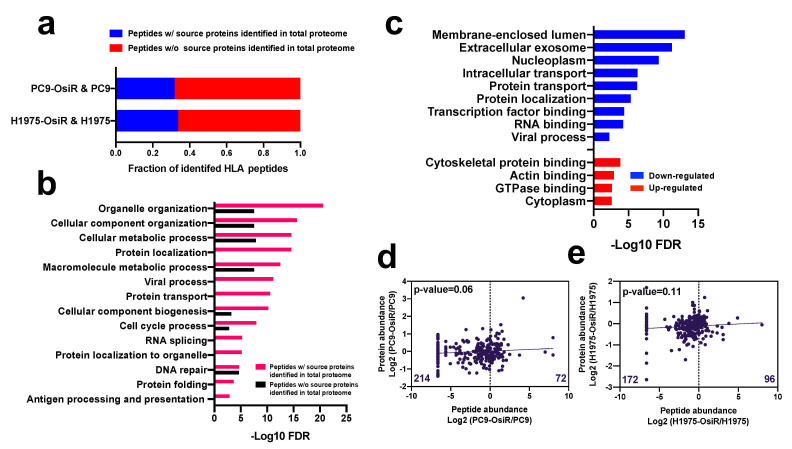
Correlation analysis of HLA class I-immunopeptide presentation and protein expression of source proteins. (**a**) Fraction of identified Class I-presented peptides with identified source proteins in the whole-cell proteome dataset. (**b**) Gene Ontology (GO) biological process annotation analysis of peptides with or without identified source proteins. (**c**) GO analysis of the source proteins of peptides with decreased (blue/down-regulated) or increased (red/up-regulated) Class I-presentation. (**d**,**e**) Linear regression analysis of total identified peptides abundance and their corresponding protein expression in PC9-OsiR/PC9 cells (**d**) and H1975-OsiR/H1975 cells (**e**). Median peptide abundance was used for the analysis if multiple peptides were derived from the same protein.

Based on our global proteomic analysis, nearly one third of the class I-presented immunopeptides were derived from proteins identified in total proteome dataset (Figure 3a). This suggests that the source proteins of most HLA class I-presented peptides have low abundance in the cellular proteome. The gene ontology (GO) analysis [31] of the peptides (by gene name) with or without identified source proteins in our proteomic profiling showed that the peptides with identified source proteins in our total proteome were more significantly involved in critical biological processes, such as metabolic process and organelle organization; importantly, many pathways were exclusively enriched in this group of peptides, such as protein localization, viral process, and protein folding (Figure 3b). Similarly, the GO analysis of the source proteins (by gene name) of the HLA Class I-presented peptides with increased or decreased Class I presentation displayed that peptides with decreased presentation were derived from genes enriched in membrane, exosome, protein localization, and viral process while those with increased presentation were enriched in cytoplasm and actin binding (Figure 3c). Endogenous proteins are degraded and presented by HLA molecules [34]; thus, we asked whether there is a correlation between the abundance of the class I-presented peptides and their source proteins. We observed no significant correlation between SILAC abundance ratios (H/L) of the Class I-presented peptides and the corresponding SILAC ratios of the source proteins (Figure 3d,e and Table S5), suggesting that the extent of Class I presentation of peptides is not just dependent on protein abundance. Interestingly, we found more Class I-presented peptides with reduced abundance in OsiR cells compared to sensitive cells. There are 214 peptides had negative log2 H/L ratio in the PC9-OsiR/PC9 SILAC experiment compared to only 72 peptides with positive values (Figure 3d). Furthermore, we observed no correlation between the source protein abundance and Class I-presented peptide abundance of proteins involved in antigen processing and presentation, protein folding, and protein localization (Figure S2). However, there were select proteins with good correlation of protein abundance and peptide presentation. For example, we observed reduction of calreticulin (CALR), protein disulfide-isomerase A6 (PDIA6) and A3 (PDIA3) in both protein expression and peptide presentation in OsiR cells. Taken together, our data shows that class I-presentation is not always proportional to protein abundance; rather peptides from proteins with very low abundance in cells may be specifically presented by HLA-class I molecules. Furthermore, there are proteins that are presented less on Class I despite increased expression in OsiR cells.

### 3.4. Quantitative Global Proteome Analysis Revealed Potential Molecular Mechanism of Reduced Antigen Presentation in Osimertinib Resistant Lung Adenocarcinoma

**Figure 4:**
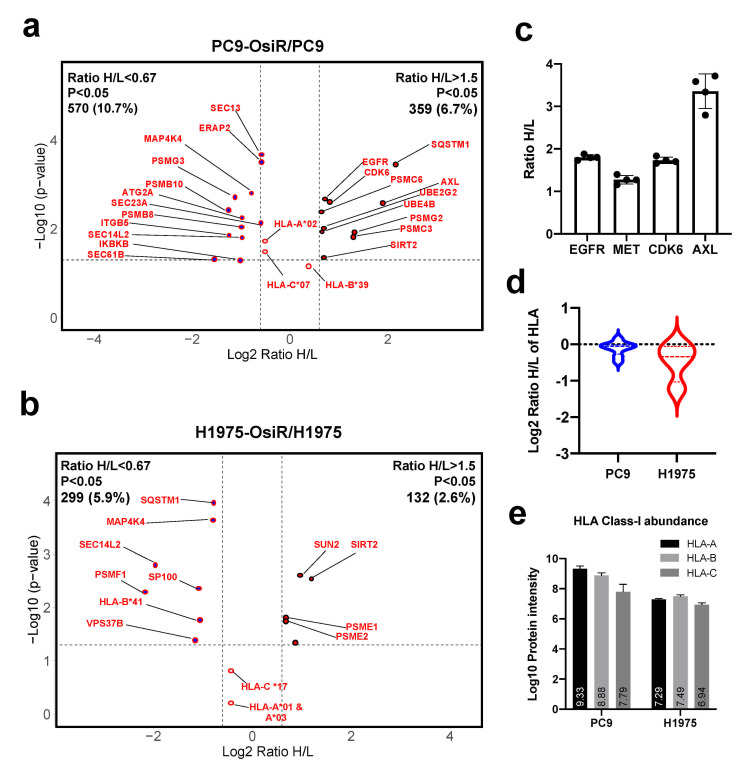
Total proteome analysis reveals the potential molecular mechanisms of overall reduced antigen presentation upon osimertinib resistance. (**a**,**b**) Volcano plots show the quantitative analysis of SILAC-labeled proteins in total proteome of PC9-OsiR/PC9 (**a**) and H1975-OsiR/H1975 lung adenocarcinoma cells (**b**). (**c**) Fold changes (Ratio H/L) of EGFR, MET, CDK6 and AXL in PC9-OsiR/PC9 cells. (**d**) Violin plot shows HLA class I protein expression in both cell lines. (**e**) Bar charts show the total protein abundance of HLA-A, HLA-B, and HLA-C in (**a**) Bar charts show the total protein abundance of HLA-A, HLA-B, and HLA-C in PC9 and H1975 cells.

Next, we sought to identify the potential mechanisms of reduced antigen presentation in OsiR cells. Using 2D offline fractionated deep whole-cell proteomics, we identified 929 (359 up- and 570 down-regulated) and 431 (132 up- and 299 down-regulated) differentially expressed proteins in PC9-OsiR and H1975-OsiR cells, respectively (Figure 4a,b and Table S1). Our data showed increased expression of EGFR, MET, CDK6, and AXL in PC9-OsiR cells (Figure 4c), and they have been recognized as key proteins involved in osimertinib resistance mechanisms [35,36,37,38]. Since HLA proteins are highly polymorphic and “shotgun” proteomics can detect limited number of unique peptides for each HLA allele, only two-digit typing can be achieved. The overall HLA class I expression was lower in OsiR cells; the decrease was more pronounced in H1975-OsiR cells compared to PC9-OsiR cells (Figure 4d). We also determined the total protein abundance of three major HLA proteins, HLA-A, HLA-B, and HLA-C. In PC9 cells, HLA-A displayed higher protein abundance compared to HLA-B and HLA-C; on the other hand, in H1975 cells, three major HLA types showed similar intensity (Figure 4e). To study the overall altered cell signaling pathways related to antigen presentation, we conducted a pathway analysis of total proteome to identify altered molecular functions of the proteins with differential expression in OsiR cells. Since the purpose of this study was to primarily investigate antigen processing and presentation, we visualized protein expression of select genes in HLA complex, protein transport, NF-κB pathway, proteasome assembly and autophagy.

#### 3.4.1. Alterations in Proteins in HLA Complex, Protein Transport, Proteases and Peptidases

**Figure 5:**
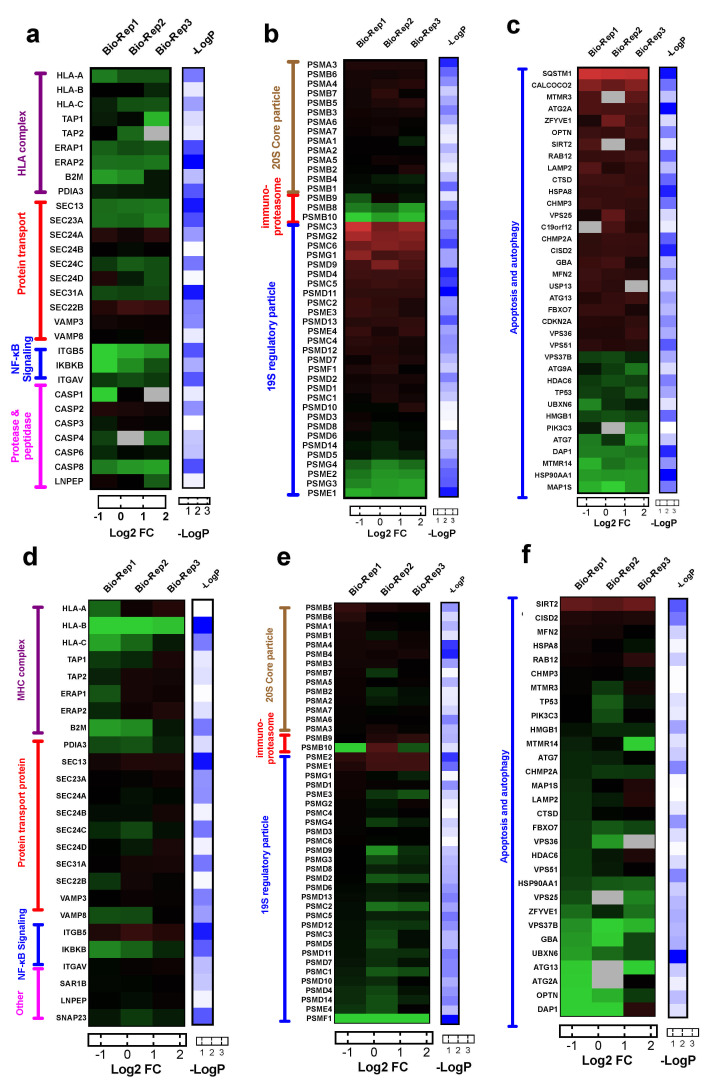
(**a**–**c**) Heatmaps of log2 ratio H/L (i.e., fold change) of proteins involved in antigen processing and presentation (**a**), proteasome assembly (**b**), autophagy and apoptosis cascade (**c**) in PC9-OsiR/PC9 cells. (**d**–**f**) Heatmaps of log2 ratio H/L (i.e., fold change) of proteins involved in antigen processing and presentation (**d**), proteasome assembly (**e**), autophagy and apoptosis cascade (**f**) in H1975-OsiR/H1975 cells. Note that −logP standards for the −log10 of one-sample t-test *p*-values.

To determine the alterations of genes involved in antigen processing and presentation via HLA class I (GO:0002474), we interrogated the expression of 25 out of 43 genes of this pathway that were identified in our whole-cell proteome dataset. Our results suggest that antigen presentation by HLA class I complex may be inhibited due to reduced expression of key components: β2 macroglobulin (B2M), antigen peptide transporter (TAP1 and TAP2) and endoplasmic reticulum aminopeptidase (ERAP1 and ERAP2) in both PC9-OsiR and H1975-OsiR cells (Figure 5a,d). Further, protein transport has been reported to play critical roles in antigen presentation [39]. Protein transport-related proteins had mixed expression changes; for example, SEC24A was up-regulated but SEC23A was significantly down-regulated in PC9-OsiR cells. Activation of NF-κB is essential to antigen presentation [40]. Notably, one of the key regulators of NF-κB signaling, inhibitor of nuclear factor kappa-B kinase subunit beta (IKBKB), had significantly reduced expression in both OsiR cells. Furthermore, we identified several down-regulated proteases in caspase group and other peptidases (e.g., Leucyl-cystinyl aminopeptidase, LNPEP) that had reduced expression in OsiR cells. LNPEP is a peptidase involved in trimming proteins in endosomes and phagosomes similar to ERAP1/2 [41].

#### 3.4.2. Proteasome Assembly

Antigen processing and presentation by Class I is dependent on the proteasome pathway [42]. Immunoproteasomes are involved in processing peptides to fit in the groove of HLA-I molecules [43]. Inhibited immunoproteasomes could potentially reduce antigen expression. We identified 47 and 46 proteins involved in proteasome assembly and interrogated their abundance ratios in PC9-OsiR/PC9 and H1975-OsiR/H1975 cells, respectively (Figure 5b,e). Most of the proteasome subunits in 20S core particle remained unchanged. Interestingly, protein expression of all three immunoproteasome subunits β1i (PSMB9), β2i (PSMB10), β5i (PSMB8) were reduced (1.4-, 2.2- and 1.6-fold, respectively) in the PC9-OsiR cells. For the 19S regulatory particle, 26S proteasome regulatory subunit 6A (PSMC3), 26S proteasome regulatory subunit 10B (PSMC6), proteasome assembly chaperone 2 (PSMG2) were upregulated, and four other subunits were downregulated in OsiR cells.

#### 3.4.3. Autophagy

Autophagy plays a critical role in metabolic homeostasis and antigen processing [44]. We also identified 36 differentially altered proteins involved in autophagy (GO:0006914), of which 24 show increased and 11 show decreased abundance in PC9-OsiR cells (Figure 5c). For instance, protein expression of calcium-binding and coiled-coil domain-containing protein 2 (CALCOCO2), an autophagy signaling activator, was increased by 2.6-fold; however, three negative regulators, sequestosome-1 (SQSTM1), NAD-dependent protein deacetylase sirtuin-2 (SIRT2) and death-associated protein 1 (DAP1), were either upregulated by 4.6 and 1.7-fold, or downregulated by 1.8-fold in PC9OsiR/PC9 experiment (Figure 5c). Similar alteration in protein abundance of a subset of autophagy proteins was observed in H1975-OsiR cells, such as for SIRT2 and DAP1 (Figure 5f).

Taken together, our results demonstrate reduced protein expression of key components of the antigen processing and presentation machinery in osimertinib resistant lung adenocarcinoma cells.

### 3.5. HLA Class I Interactome Profiling in Osimertinib Sensitive and Resistant Cells

**Figure 6:**
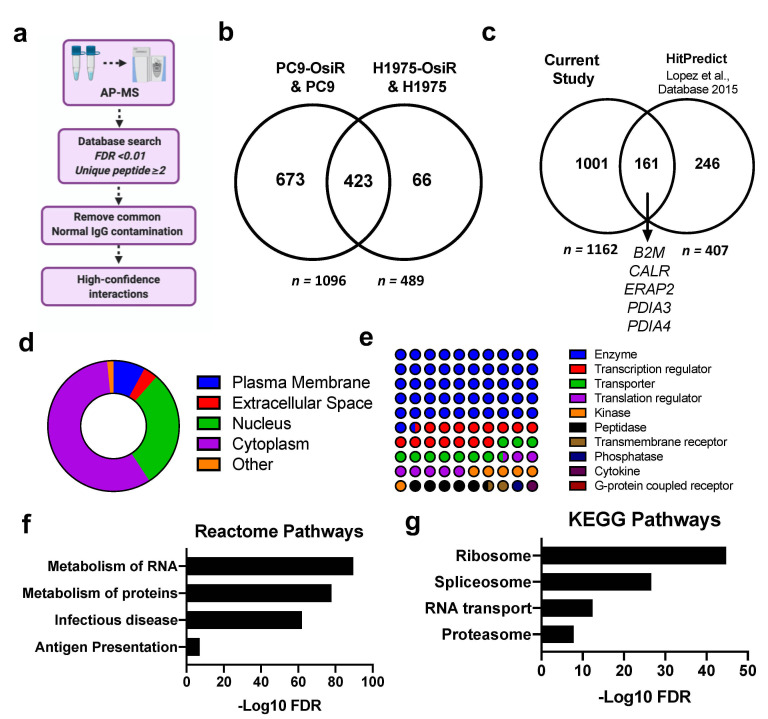
Large-scale affinity purification-mass spectrometry (AP-MS) profiling uncovers direct or indirect interaction partners of HLA class I molecules. (**a**) Schema of the informatic pipeline to retrieve high-confidence interactions (HCIs) of HLA Class I. (**b**) Venn diagram shows the overlapping HCIs between PC9-OsiR/PC9 and H1975-OsiR/H1975 experiments. (**c**) Venn diagram shows the overlapping HCIs of current study and known partners reported in databases. (**d**) The subcellular localization of Class I interacting proteins. (**e**) Dot plot shows the primary molecular functions of class I interacting proteins. (**f**,**g**) Pathway analysis of identified HCIs using KEGG (**f**) and Reactome database (**g**).

To comprehensively study the molecular mechanisms of potentially inhibited antigen processing and presentation, we performed SILAC-based HLA Class I quantitative interactome analysis. Our workflow first separated Class I presented immunopeptides by low percentage organic buffer (i.e., 30% acetonitrile), then subsequently eluted the HLA protein complex using a higher percentage organic buffer (i.e., 80% acetonitrile). To determine the high-confidence interactions (HCIs), the AP-MS identified Class I-interacting proteins underwent several stringent informatic filters: (a) false discovery rate (FDR) of each protein < 0.01 upon database search; (b) each protein identified with no less than two unique peptides; (c) common normal IgG contaminations were removed using CRAPome (crapome.org) (Figure 6a). In total, we identified 1096 and 489 HCIs from PC9/PC9-OsiR and H1975/H1975-OsiR SILAC experiments, respectively. Strikingly, 87% (423/489) HCIs identified in H1975 overlapped with the ones identified in PC9 cells (Figure 6b). To validate our datasets with previous reports, we leveraged HitPredict database compiling multiple large-scale databases (e.g., BioGRID, IntAct, BioPlex) to match our HCIs with known HLA-A, HLA-B, HLA-C interaction partners (*n* = 407) (Table S6) [45]. We identified 40% (161/407) of the known HLA interactions, including B2M, CALR, ERAP2, PDIA3, and PDIA4. We identified ~1000 novel Class I-interacting proteins (Figure 6c). The subcellular component analysis displayed that ~60% of the HCIs are primarily cytosolic proteins, ~30% nuclear, and a small fraction cell membrane proteins (Figure 6d). Majority of HLA Class I-interacting proteins identified in our dataset reside in the cytosol, including proteins in the proteasome, ribosome, lysosome, and endoplasmic reticulum. The cellular function analysis show that more than half of the HCIs are enzymes, kinases, and peptidases. Transcription factors and transporters comprised ~20% of total HCIs. A very small portion belonged to the transmembrane receptors (Figure 6e). The pathway analysis of total HLA interactome were performed using KEGG and Reactome databases (Figure 6f,j) where ribosome, proteasome, RNA transport, metabolism of proteins, and antigen presentation pathways were significantly enriched.

**Figure 7:**
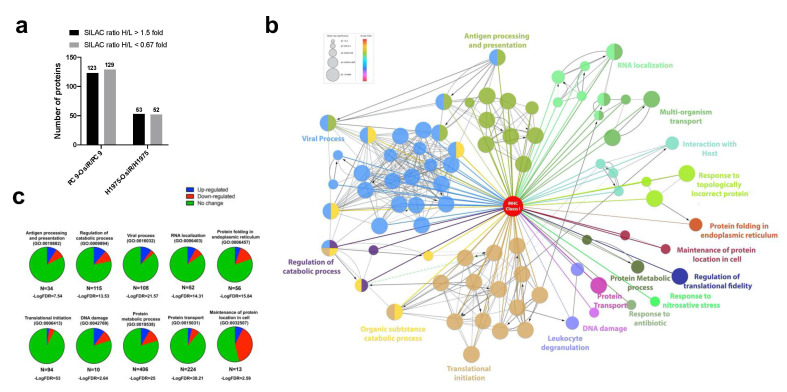
Altered interactome network analysis reveals possible signaling pathways involved in antigen processing and presentation upon osimertinib resistance. (**a**) SILAC-based quantitative interactome analysis unveils the up- and down-regulated association with HLA class I complex. (**b**) HLA class I-interactome network was generated using selected HCIs in PC9-OsiR/PC9 experiment. (**c**) Pie charts show the number of proteins differentially interacting with HLA class I complex in top 10 select GO annotated signaling pathways.

Next, we quantified the HCIs to explore the potential role of altered Class I-interaction in antigen processing and presentation in OsiR cells. The statistically significant normalized SILAC ratio was used to determine altered (cutoff = 1.5 or 0.67) interaction with HLA Class I proteins; ~20% of the total interactome (10% increased and 10% decreased) were significantly altered in OsiR cells (Figure 7a). To visualize the relationships between the identified HCIs, we leveraged ClueGo and CluePedia databases to generate, to date, the largest Class I protein-protein interaction network using Cystoscape informatic package (Figure 7b,c and Figure S3a). As expected, the network contained antigen processing and presentation and viral process. The network also contained proteins involved in protein folding in endoplasmic reticulum, maintenance of protein localization, regulation of translational fidelity, protein transport, RNA localization, protein metabolic process, DNA damage, and regulation of catabolic process. Our quantitative Class I interactome analysis identified biological processes that are likely altered in OsiR cells, including but not limited to protein folding, DNA damage and maintenance of protein localization. The 22 proteasome proteins that interacted with HLA Class I molecules showed a decreased association trend (15 proteins with log2 ratio < 0 versus 7 with a Log2 ratio > 0) in the PC9-OsiR cells compared to the sensitive cells (Figure S3b).

**Figure 8:**
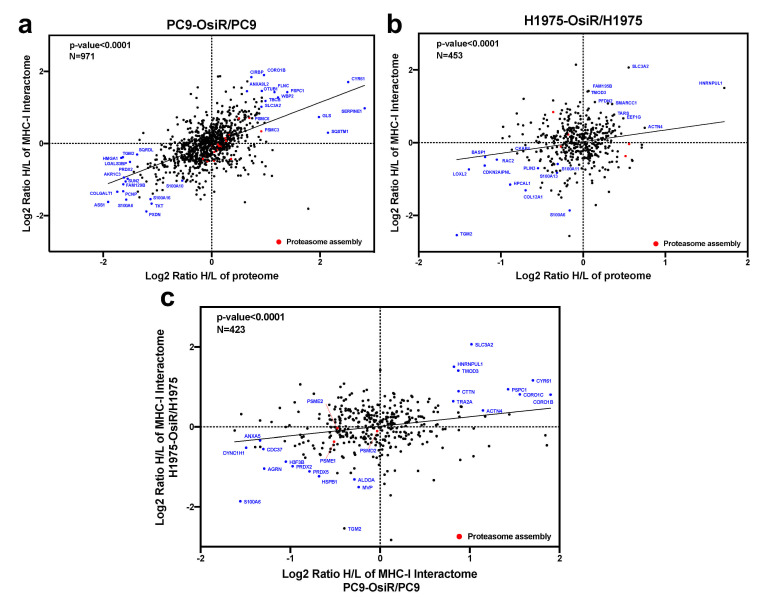
Correlation analysis of proteome and interactome reveals key functional proteins in altered antigen presentation signaling in OsiR cells. (**a**,**b**) Correlation analysis of proteomic and interactomic datasets in PC9-OsiR/PC9 (**a**) and H1975-OsiR/H1975 lung adenocarcinoma cells (**b**). (**c**) Correlation analysis of the HCIs identified both in PC9-OsiR/PC9 and H1975-OsiR/H1975 experiments.

Further, we leveraged the total proteome and interactome to unveil key regulatory proteins that were inhibited or activated in OsiR cells by both unbiased analyses. The intracellular proteome positively correlated with class I interactome (*p* < 0.0001) in both PC9/PC9-OsiR and H1975/H1975-OsiR lung adenocarcinoma cellular pairs. Notably, our data suggests S100 proteins (e.g., S100A6, S100A10, S100A16) that are calcium-dependent binding proteins, were both down-regulated in protein abundance and association with HLA complex. In contrast, specific proteins, such as 4F2 cell-surface antigen heavy chain (SLC3A2), an amino acid transport protein was up regulated in protein expression and showed stronger binding to HLA Class I (Figure 8a,b). The differential changes of HLA interactome comparing OsiR to parental cells in both cell lines were significantly correlated (linear regression *p* < 0.0001), which provided higher confidence in our observations (Figure 8c). Furthermore, three proteasomal proteins (PSME1, PSME2, PSMD2) exhibited reduced interaction with HLA complex in both PC9-OsiR and H1975-OsiR. Taken together, our findings of the novel HLA Class I interacting proteins provide evidence of significantly alteration of several potential cell-signaling pathways in OsiR cells that have relevance in antigen processing and presentation (e.g., protein folding, proteasomal degradation, amino acid uptake, and maintenance of protein localization).

### 3.6. Integrated Pathway Analysis of Significantly Altered Proteins

**Figure 9:**
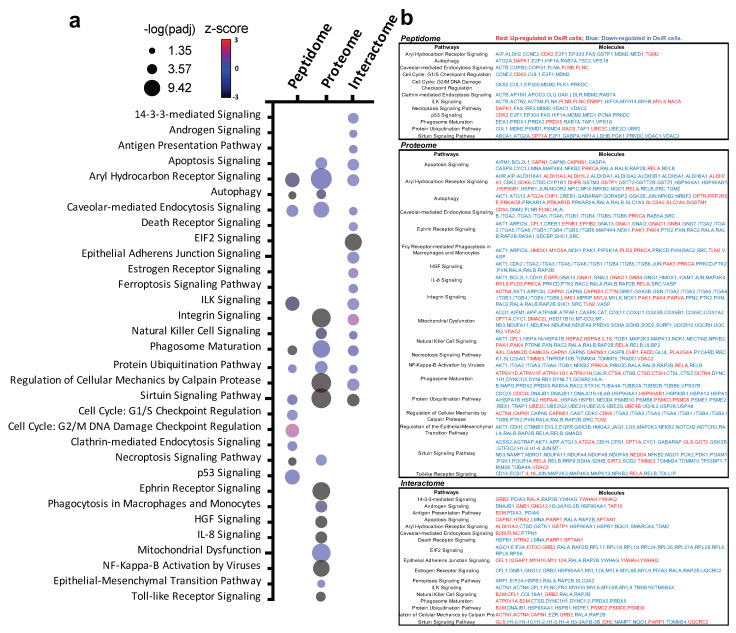
Intergard pathway analysis of significantly altered proteins. (**a**) Ingenuity Pathway Analysis (IPA) of significantly altered proteins (Log2 Ratio H/L > |0.6|) identified in peptidome, proteome, interactome in at least in one OsiR cell line, PC9-OsiR/PC9 and/or H1975-OsiR/H1975. Noted that only significantly enriched pathways were shown (*p*-value adjusted < 0.05); positive z-score indicates activated pathways, and negative z-score indicates inhibited pathways. (**b**) Table chart shows the gene names involved in significant enriched pathways in peptidome, proteome and interactome, respectively. Red coded genes indicate up-regulated/presented in OsiR, and blue coded genes indicate down-regulated/presented in OsiR.

To reveal biological mechanisms of altered antigen processing and presentation in OsiR, we conducted integrated pathway analyses of significantly altered proteins in peptidome (source protein of identified peptides), whole-cell proteome, and HLA Class I interactome (Figure 9a,b). We selected 32 significantly enriched pathways related to immunology and cancer biology. Based on the fold changes (i.e., ratio H/L, Osi-R/sensitive cells) of proteins in those pathways, z-scores of each pathway were calculated where positive values indicate activation and negative values indicate inhibition. Strikingly, many pathways were inhibited in OsiR cells, such as autophagy, phagosome maturation, NF-κB signaling, IL-8 signaling, and sirtuin pathway; in contrast, several pathways were activated, such as cell cycle, p53 and ubiquitination. Significantly altered proteins of the enriched pathways from the peptidome, total proteome, and Class I-interactome datasets (Figure 9b), providing a rich resource to further investigate altered antigen presentation in osimertinib resistance.

## 4. Discussion

**Figure 10:**
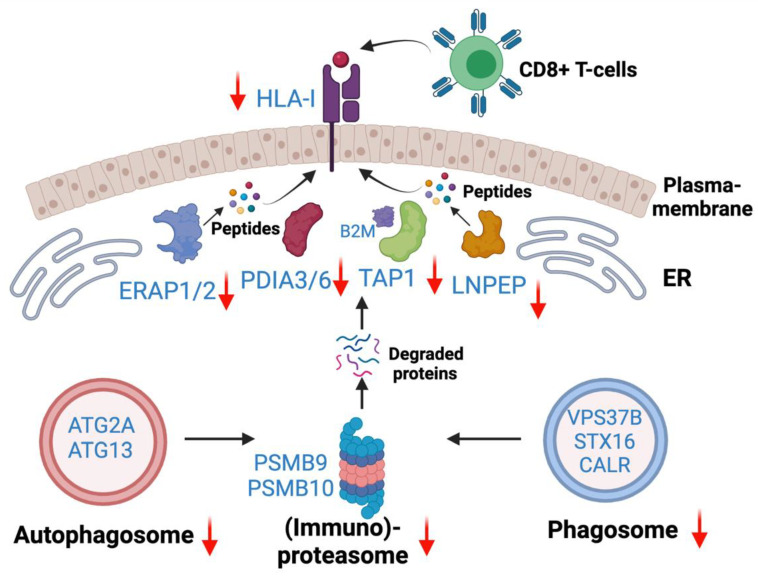
Antigen generation and HLA Class—I associated antigen presentation signaling pathway. Down-regulated autophagosome, immunoproteasome, phagosome were main avenues to generate degraded protein (e.g., antigen). Down-regulated HLA-I complex and peptidases contribute to reduced antigen presentation in OsiR.

Integration of existing ICI therapies as well as novel immunotherapies in EGFR mutant lung adenocarcinoma are unmet needs. However, the mechanisms of inadequate responses to ICI therapies or increased toxicities in combination with EGFR targeted therapies are inadequately understood. To that end, this study is a comprehensive characterization of altered Class I-mediated antigen presentation and Class I interaction that will enable further studies to understand the underlying mechanisms of poor ICI therapy or identify novel targets for precision immunotherapies. The present study, for the first time, revealed the overall reduced HLA antigen presentation in osimertinib resistant human lung adenocarcinoma. Quantitative total proteome data revealed reduced protein expression among proteins in the core HLA complex machinery, immunoproteasome, and several key peptidases and proteases involved in antigen processing and presentation. Based on the differentially expressed proteins and altered pathways observed in this study, we provide a schema showing potential molecular mechanisms of immune escape in Osimertinib resistant cells (Figure 10). OsiR cells have reduced immunoproteasome proteins compared to sensitive cells (e.g., PSMB9 and PSMB10). Intracellular mis-spliced or normal proteins are degraded in the immunoproteasome. Studies have demonstrated that autophagosome and phagosome are the sources of HLA antigens [46]. OsiR cells have significantly inhibited autophagy (e.g., ATG2A, ATG13) and phagosome (e.g., VSP37B and STX16) proteins. Furthermore, the degraded or truncated products are transported by TAP1 and subsequently digested by aminopeptidases (e.g., ERAP1/2 and LNPEP) and presented by PDIA3/6, B2M and HLA Class I complex to CD8+ T lymphocytes. We demonstrated that all these key HLA presentation components were significantly downregulated. Interestingly, there was increased association of B2M with Class I proteins in OsiR cells, the significance of which remains to be determined. Overall, this study demonstrated some key mechanisms of potentially reduced antigen processing and presentation upon EGFR TKI resistance in lung cancer.

The Class I-presented immunopeptidome identified in this dataset is a unique resource for the demonstration of actual peptides presented by Class I proteins in EGFR mutant lung adenocarcinoma cells. Nearly all quantified peptides were in the 8–14 mer range (8–14 aminoacids in length) which is the dominant peptide length fitting the HLA Class I-binding grooves; as expected, 9 mer peptides were the most frequently identified peptides (Figure 1h,i). Leveraging a well-established T cell epitope prediction algorithm (i.e., NetMHCpan), a majority of identified peptides were found to be predicted binders to at least one HLA allele in the corresponding cell line (Figure 2b). Motif analysis of the identified peptides demonstrated similarity of the motifs of the identified peptides with the corresponding Class I monoallelic-presented epitopes in IEDB database (Figure 2d,e), strengthening the validity of this dataset. Rosenthal and colleagues reported reduced neoantigen (tumor specific antigen) load during lung cancer evolution, providing a route to immune evasion [47]. Clinical outcome to immunotherapies associates with neoantigen load [48]. Our findings suggest that not only loss of neoantigens, but also reduced global landscape of antigen presentation may induce immune escape in EGFR mutant lung adenocarcinoma. We did not observe significant association between protein expression of source proteins and Class I -presented peptides (Figure 3d,e) in contrast to a reported study where strong correlation was observed between protein abundance on antigen presentation [49]. This indicates that epitope presentation is not always dependent on protein abundance. We posit that antigen processing and presentation is tightly regulated and often antigen specific. Indeed, while the global Class I presented peptides did not correlate with source protein expression, specific targets such as the CALR, PDIA3, PDIA6 had reduced expression as well as Class I presentation in OsiR cells.

This study, for the first time to our knowledge, examined the Class I-presented immunopeptidome and Class I interactome in the same experiment. We interrogated the direct and indirect interacting proteins of Class I proteins and quantified the level of interaction in osimertinib sensitive and resistant lung adenocarcinoma cells. After removing the low-confident and non-specific binding with several stringent criteria, we identified large fraction of HLA HCIs overlapped between PC9 and H1975 cell lines. Importantly, we identified 1162 novel HLA class I interaction partners that have not been reported before. The pathway analysis and interaction network displayed multiple differentially regulated signaling pathways correlated with those in total proteomic dataset, such as protein folding, apoptosis, and ubiquitination (Figure 7b). The amino acid transporter, SLC3A2, also known as CD98 heavy chain (CD98hc) had increased expression in intracellular proteome and increased Class I interaction in HLA interactome datasets in both cell lines (Figure 8a,b). CD98hc activates T-cell clonal expansion to enable adaptive immunity [50,51]. Studies also have shown that SLC3A2 is overexpressed in lung cancer and is associated with poor prognosis [52]. Our finding indicates SLC3A2 may play critical role in antigen processing and presentation.

Our integrated pathway analysis demonstrated that source of antigen could be affected by OsiR: (a) Immunoproteasome proteins (e.g., PSMB8, PSMB9 and PSMB10) have reduced expression in OsiR cells. The immunoproteasome is a fast responder to interferon gamma (IFN-γ) signaling which stimulates overall antigen presentation [53,54]. Mice lacking all three immunoproteasome proteins have impaired MHC Class I antigen presentation [55]. (b) Many key elements in autophagy are down-regulated in OsiR compared to proteasome-mediated protein degradation, autophagy results in lysosome-mediated protein degradation, commonly eliminating long-lived proteins and processing of short-lived proteins (e.g., misfolded proteins), providing epitopes for both class I and class II molecules [56,57]. (c) Caspases, a group of proteases, (e.g., CASP4 and CASP8), have been reported to mediate protein degradation in a caspase-dependent manner and stimulate CD8 T-cell activation via recognizing “self” antigens [58,59]. CASP3, CASP6, and CASP8 had significantly reduced abundance in PC9-OsiR cells. (d) Phagosome signaling was inhibited in OsiR cells. Phagocytosis of mis-spliced or mutated proteins can generate the epitopes presented by HLA class I molecules via “cross-presentation” [60]. Furthermore, in our dataset, multiple key components in antigen processing and presentation have reduced expression in OsiR cells: (a) HLA core complex (e.g., HLA-B, TAP1). TAP-deficient cells reduce the cell surface HLA expression [61]. (b) Several aminopeptidases are downregulated in OsiR cells, such as ERAP1/2 and LNPEP. These proteins are major enzymes that trim precursor peptides into desired shorter peptides (usually 8–14 mer) for Class I presentation [62,63].

We acknowledge a few of caveats in this study: (a) Although SILAC labeled native immunopeptides represent the majority of identified peptides, those without both a lysine or an arginine were not labeled and hence, could not be quantified; we could still quantify more than 60 % of identified class I presented peptides (b) our innovative Class I-presented immunopeptides and HLA complex separation pipeline from the same experiment could result in the low hydrophobic HLA class I HCIs to be eluted off with the Class I-presented immunopeptides using 30% ACN buffer and hence, not identified; (c) due to the large amount of required cell martial (200 million cells/replicate), we leveraged best known nonspecific binding proteins in the CRAPome database; several replicates using isotype control beads might have been better negative controls; (d) in contrast to tryptic peptides, native peptides generated in vivo may exhibit poor ionization and detection in mass spectrometry [13].

## 5. Conclusions

In conclusion, we provide evidence of possible global inhibition of HLA peptide processing and presentation upon osimertinib resistance in EGFR mutant lung adenocarcinoma. Reduced expression and/or interaction of the HLA Class I complex proteins potentially reduce Class I antigen presentation upon EGFR TKI resistance. Suppressed immunoproteasome and autophagy cascades that are known to influence antigen processing and presentation are likely drivers of immune evasion mechanisms in EGFR mutant lung cancer. The extensive dataset of the Class I-presented immunopeptidome, Class I interactome, and total proteome upon osimertinib resistance has the potential to generate novel targets for immunotherapy in EGFR mutant lung cancer in future studies.

## Data Availability

The raw MS data presented in this study are openly available on Synapse with access ID: syn25915977 (https://www.synapse.org/#!Synapse:syn25915975/files) and it has been publicly available since 3 October 2021.

## Supplementary Materials

The following are available online at https://www.mdpi.com/article/10.3390/cancers13194977/s1, Figure S1: Cell line sources and motif analysis of HLA Class I immunopeptidome. (a) Cell line sources of PC9 and H1975 with accession ID. (b) The correlations among biological replicates of PC9/PC9-OsiR immunopeptidome. (c) The motif analysis of corresponding monoallelic HLA allele binding peptides reported in IEDB. The HLA alleles expressed in PC9-OsiR and PC9 cells are HLA-A*02:06, HLA-A*24:02, HLA-B*39:01, HLA-Cw*07 and HLA-Cw*03. (d) The binding motif of 9 mer peptides identified in H1975-OsiR/H1975 cells. (e) The motif analysis of corresponding monoallelic HLA allele binding peptides reported in IEDB. The HLA alleles expressed in H1975-OsiR and H1975 cells are HLA-A*01:01, HLA-A*03:01, HLA-B*41:01and HLA-Cw*17, Figure S2: Correlation of HLA Class I immunopeptides and their source proteins in (a–c) genes involved in antigen processing and presentation, protein folding and protein localization pathways in PC9-OsiR/PC9 cells and (d–f) genes involved in antigen processing and presentation, protein folding and protein localization pathways in H1975-OsiR/H1975 cells, Figure S3: (a) Interactome network visualization of HLA Class I interacting partners in H1975-OsiR&H1975 cells. (b) The differentially altered association of proteasomal proteins with HLA complex in PC9-OsiR and PC9 cells, Table S1: Total proteome identification and quantification of SILAC labeled PC9-OsiR and PC9 and H1975-OsiR and H1975, Table S2: HLA Class I-presented immunopeptidome identification and quantification of SILAC labeled PC9-OsiR and PC9 and H1975-OsiR and H1975, Table S3: HLA Class I interactome identification and quantification of SILAC labeled PC9-OsiR and PC9 and H1975-OsiR and H1975, Table S4: NetMHCpan 4.0 prediction of identified 9–11 mer peptides, Table S5: Correlation of HLA Class I-presented immunopeptidome and their source proteins, Table S6: Known HLA Class I interacting proteins previously reported in HitPredict.

## References

[1] Dong Z.-Y., Zhang J.-T., Liu S.-Y., Su J., Zhang C., Xie Z., Zhou Q., Tu H.-Y., Xu C.-R., Si-Yang L. (2017). EGFR mutation correlates with uninflamed phenotype and weak immunogenicity, causing impaired response to PD-1 blockade in non-small cell lung cancer. OncoImmunology.

[2] Hastings K., Yu H., Wei W., Sanchez-Vega F., DeVeaux M., Choi J., Rizvi H., Lisberg A., Truini A., Lydon C. (2019). EGFR mutation subtypes and response to immune checkpoint blockade treatment in non-small-cell lung cancer. Ann. Oncol..

[3] Schmid S., Li J., Leighl N.B. (2020). Mechanisms of osimertinib resistance and emerging treatment options. Lung Cancer.

[4] Lazzari C., Gregorc V., Karachaliou N., Rosell R., Santarpia M. (2020). Mechanisms of resistance to osimertinib. J. Thorac. Dis..

[5] Lee C.K., Man J., Lord S.J., Links M., Gebski V., Mok T., Yang J.C.-H. (2017). Checkpoint Inhibitors in Metastatic EGFR- Mutated Non–Small Cell Lung Cancer—A Meta-Analysis. J. Thorac. Oncol..

[6] Ahn M.-J., Sun J.-M., Lee S.-H., Ahn J.S., Park K. (2017). EGFR TKI combination with immunotherapy in non-small cell lung cancer. Expert Opin. Drug Saf..

[7] Liang H., Liu X., Wang M. (2018). Immunotherapy combined with epidermal growth factor receptor-tyrosine kinase inhibitors in non-small-cell lung cancer treatment. OncoTargets Ther..

[8] Yang J.C.-H., Shepherd F.A., Kim D.-W., Lee G.-W., Lee J.S., Chang G.-C., Lee S.S., Wei Y.-F., Lee Y.G., Laus G. (2019). Osimertinib Plus Durvalumab versus Osimertinib Monotherapy in EGFR T790M–Positive NSCLC following Previous EGFR TKI Therapy: CAURAL Brief Report. J. Thorac. Oncol..

[9] Akbay E., Koyama S., Carretero J., Altabef A., Tchaicha J.H., Christensen C.L., Mikse O.R., Cherniack A.D., Beauchamp E.M., Pugh T. (2013). Activation of the PD-1 Pathway Contributes to Immune Escape in EGFR-Driven Lung Tumors. Cancer Discov..

[10] Vanneman M., Dranoff G. (2012). Combining immunotherapy and targeted therapies in cancer treatment. Nat. Rev. Cancer.

[11] Chen N., Fang W., Zhan J., Hong S., Tang Y., Kang S., Zhang Y., He X., Zhou T., Qin T. (2015). Upregulation of PD-L1 by EGFR Activation Mediates the Immune Escape in EGFR-Driven NSCLC: Implication for Optional Immune Targeted Therapy for NSCLC Patients with EGFR Mutation. J. Thorac. Oncol..

[12] Zhang X., Qi Y., Zhang Q., Liu W. (2019). Application of mass spectrometry-based MHC immunopeptidome profiling in neoantigen identification for tumor immunotherapy. Biomed. Pharmacother..

[13] Abelin J., Keskin D.B., Sarkizova S., Hartigan C.R., Zhang W., Sidney J., Stevens J., Lane W., Zhang G.L., Eisenhaure T.M. (2017). Mass Spectrometry Profiling of HLA-Associated Peptidomes in Mono-allelic Cells Enables More Accurate Epitope Prediction. Immunity.

[14] Bassani-Sternberg M., Chong C., Guillaume P., Solleder M., Pak H.S., Gannon P.O., Kandalaft L.E., Coukos G., Gfeller D. (2017). Deciphering HLA-I motifs across HLA peptidomes improves neo-antigen predictions and identifies allo-stery regulating HLA specificity. bioRxiv.

[15] Qi Y.A., Maity T.K., Cultraro C.M., Misra V., Zhang X., Ade C., Gao S., Milewski D., Nguyen K.D., Ebrahimabadi M.H. (2021). Proteogenomic Analysis Unveils the HLA Class I-Presented Immunopeptidome in Melanoma and EGFR-Mutant Lung Adenocarcinoma. Mol. Cell. Proteom..

[16] Bourdetsky D., Schmelzer C., Admon A. (2014). The nature and extent of contributions by defective ribosome products to the HLA peptidome. Proc. Natl. Acad. Sci. USA.

[17] Milner E., Gutter-Kapon L., Bassani-Sternberg M., Barnea E., Beer I., Admon A. (2013). The Effect of Proteasome Inhibition on the Generation of the Human Leukocyte Antigen (HLA) Peptidome. Mol. Cell. Proteom..

[18] Zhang X., Maity T., Kashyap M.K., Bansal M., Venugopalan A., Singh S., Awasthi S., Marimuthu A., Jacob H.K.C., Belkina N. (2017). Quantitative Tyrosine Phosphoproteomics of Epidermal Growth Factor Receptor (EGFR) Tyrosine Kinase Inhibi-tor-treated Lung Adenocarcinoma Cells Reveals Potential Novel Biomarkers of Therapeutic Response. Mol. Cell. Proteom..

[19] Zhang X., Nguyen K.D., Rudnick P.A., Roper N., Kawaler E., Maity T.K., Awasthi S., Gao S., Biswas R., Venugopalan A. (2019). Quantitative Mass Spectrometry to Interrogate Proteomic Heterogeneity in Metastatic Lung Adenocarcinoma and Validate a Novel Somatic Mutation CDK12-G879V. Mol. Cell. Proteom..

[20] Zhang X., Maity T.K., Ross K.E., Qi Y., Cultraro C.M., Bahta M., Pitts S., Keswani M., Gao S., Nguyen K.D.P. (2021). Alterations in the Global Proteome and Phosphoproteome in Third Generation EGFR TKI Resistance Reveal Drug Targets to Circumvent Resistance. Cancer Res..

[21] Tran N.H., Qiao R., Xin L., Chen X., Liu C., Zhang X., Shan B., Ghodsi A., Li M. (2018). Deep learning enables de novo peptide sequencing from data-independent-acquisition mass spectrometry. Nat. Methods.

[22] Boegel S., Löwer M., Schäfer M., Bukur T., De Graaf J., Boisguérin V., Türeci Ö., Diken M., Castle J.C., Sahin U. (2012). HLA typing from RNA-Seq sequence reads. Genome Med..

[23] Mellacheruvu D., Wright Z., Couzens A.L., Lambert J.-P., A St-Denis N., Li T., Miteva Y.V., Hauri S., E Sardiu M., Low T.Y. (2013). The CRAPome: A contaminant repository for affinity purification–mass spectrometry data. Nat. Methods.

[24] Colaert N., Helsens K., Martens L., Vandekerckhove J., Gevaert K. (2009). Improved visualization of protein consensus sequences by iceLogo. Nat. Methods.

[25] Jurtz V.I., Paul S., Andreatta M., Marcatili P., Peters B., Nielsen M. (2017). NetMHCpan-4.0: Improved Peptide–MHC Class I Interaction Predictions Integrating Eluted Ligand and Peptide Binding Affinity Data. J. Immunol..

[26] Juhász A., Haraszi R., Maulis C. (2015). ProPepper: A curated database for identification and analysis of peptide and immune-responsive epitope composition of cereal grain protein families. Database.

[27] Tyanova S., Temu T., Sinitcyn P., Carlson A., Hein M.Y., Geiger T., Mann M., Cox J. (2016). The Perseus computational platform for comprehensive analysis of (prote)omics data. Nat. Methods.

[28] Fabregat A., Sidiropoulos K., Viteri G., Forner-Martinez O., Marin-Garcia P., Arnau V., D’Eustachio P., Stein L., Hermjakob H. (2017). Reactome pathway analysis: A high-performance in-memory approach. BMC Bioinform..

[29] Krämer A., Green J., Pollard J., Tugendreich S. (2013). Causal analysis approaches in Ingenuity Pathway Analysis. Bioinformatics.

[30] Huang D.W., Sherman B.T., Lempicki R.A. (2009). Systematic and integrative analysis of large gene lists using DAVID bioinformatics resources. Nat. Protoc..

[31] Ashburner M., Ball C.A., Blake J., Botstein D., Butler H., Cherry J.M., Davis A.P., Dolinski K., Dwight S.S., Eppig J.T. (2000). Gene Ontology: Tool for the unification of biology. Nat. Genet..

[32] Ogata H., Goto S., Sato K., Fujibuchi W., Bono H., Kanehisa M. (1999). KEGG: Kyoto Encyclopedia of Genes and Genomes. Nucleic Acids Res..

[33] Shannon P., Markiel A., Ozier O., Baliga N., Wang J.T., Ramage D., Amin N., Schwikowski B., Ideker T. (2003). Cytoscape: A Software Environment for Integrated Models of Biomolecular Interaction Networks. Genome Res..

[34] Rock K.L., Reits E., Neefjes J. (2016). Present Yourself! By MHC Class I and MHC Class II Molecules. Trends Immunol..

[35] Nukaga S., Yasuda H., Tsuchihara K., Hamamoto J., Masuzawa K., Kawada I., Naoki K., Matsumoto S., Mimaki S., Ikemura S. (2017). Amplification of EGFR Wild-Type Alleles in Non–Small Cell Lung Cancer Cells Confers Acquired Resistance to Mutation-Selective EGFR Tyrosine Kinase Inhibitors. Cancer Res..

[36] Yu H.A., Arcila M.E., Rekhtman N., Sima C.S., Zakowski M.F., Pao W., Kris M., Miller V.A., Ladanyi M., Riely G.J. (2013). Analysis of Tumor Specimens at the Time of Acquired Resistance to EGFR-TKI Therapy in 155 Patients with EGFR-Mutant Lung Cancers. Clin. Cancer Res..

[37] Le X., Puri S., Negrao M., Nilsson M.B., Robichaux J.P., A Boyle T., Hicks J.K., Lovinger K.L., Roarty E.B., Rinsurongkawong W. (2018). Landscape of EGFR-Dependent and -Independent Resistance Mechanisms to Osimertinib and Continuation Therapy Beyond Progression in EGFR-Mutant NSCLC. Clin. Cancer Res..

[38] Taniguchi H., Yamada T., Wang R., Tanimura K., Adachi Y., Nishiyama A., Tanimoto A., Takeuchi S., Araujo L.H., Boroni M. (2019). AXL confers intrinsic resistance to osimertinib and advances the emergence of tolerant cells. Nat. Commun..

[39] Adiko A.C., Ebabdor J., Martínez E.E., Guermonprez P., Esaveanu L. (2015). Intracellular Transport Routes for MHC I and Their Relevance for Antigen Cross-Presentation. Front. Immunol..

[40] Zhou Y., Bastian I.N., Long M.D., Dow M., Li W., Liu T., Ngu R.K., Antonucci L., Huang J.Y., Phung Q.T. (2021). Activation of NF-kappaB and p300/CBP potentiates cancer chemoimmunotherapy through induction of MHC-I antigen presentation. Proc. Natl. Acad. Sci. USA.

[41] Saveanu L., van Endert P. (2012). The role of insulin-regulated aminopeptidase in MHC class I antigen presentation. Front. Immunol..

[42] Gaczynska M., Rock K.L., Goldberg A.L. (1993). Role of Proteasomes in Antigen Presentation. Enzym. Protein.

[43] Ferrington D.A., Gregerson D.S. (2012). Immunoproteasomes: Structure, function, and antigen presentation. Prog. Mol. Biol. Transl. Sci..

[44] Van Kaer L., Parekh V.V., Postoak J.L., Wu L. (2017). Role of autophagy in MHC class I-restricted antigen presentation. Mol. Immunol..

[45] López Y., Nakai K., Patil A. (2015). HitPredict version 4: Comprehensive reliability scoring of physical protein–protein interactions from more than 100 species. Database.

[46] Cadwell K. (2016). Crosstalk between autophagy and inflammatory signalling pathways: Balancing defence and homeostasis. Nat. Rev. Immunol..

[47] RRosenthal R., Cadieux E.L., Salgado R., Al Bakir M., Moore D.A., Hiley C.T., Lund T., Tanic M., Reading J., The TRACERx consortium (2019). Neoantigen-directed immune escape in lung cancer evolution. Nature.

[48] Lauss M., Donia M., Harbst K., Andersen R., Mitra S., Rosengren F., Salim M., Vallon-Christersson J., Törngren T., Kvist A. (2017). Mutational and putative neoantigen load predict clinical benefit of adoptive T cell therapy in melanoma. Nat. Commun..

[49] Bassani-Sternberg M., Pletscher-Frankild S., Jensen L.J., Mann M. (2015). Mass Spectrometry of Human Leukocyte Antigen Class I Peptidomes Reveals Strong Effects of Protein Abundance and Turnover on Antigen Presentation. Mol. Cell. Proteom..

[50] Cantor J.M., Ginsberg M.H. (2012). CD98 at the crossroads of adaptive immunity and cancer. J. Cell Sci..

[51] Cantor J., Slepak M., Ege N., Chang J., Ginsberg M.H. (2011). Loss of T Cell CD98 H Chain Specifically Ablates T Cell Clonal Expansion and Protects from Autoimmunity. J. Immunol..

[52] Kaira K., Oriuchi N., Imai H., Shimizu K., Yanagitani N., Sunaga N., Hisada T., Kawashima O., Kamide Y., Ishizuka T. (2009). CD98 Expression Is Associated with Poor Prognosis in Resected Non-Small-Cell Lung Cancer with Lymph Node Metastases. Ann. Surg. Oncol..

[53] Groettrup M., Standera S., Stohwasser R., Kloetzel P.M. (1997). The subunits MECL-1 and LMP2 are mutually required for incorporation into the 20S proteasome. Proc. Natl. Acad. Sci. USA.

[54] Heink S., Ludwig D., Kloetzel P.M., Krüger E. (2005). IFN-gamma-induced immune adaptation of the proteasome system is an accelerated and transient response. Proc. Natl. Acad. Sci. USA.

[55] Kincaid E.Z., Che J.W., York I., Escobar H., Reyes-Vargas E., Delgado J.C., Welsh R.M., Karow M.L., Murphy A., Valenzuela D.M. (2011). Mice completely lacking immunoproteasomes show major changes in antigen presentation. Nat. Immunol..

[56] Kocaturk N.M., Gozuacik D. (2018). Crosstalk Between Mammalian Autophagy and the Ubiquitin-Proteasome System. Front. Cell Dev. Biol..

[57] Crotzer V.L., Blum J.S. (2009). Autophagy and Its Role in MHC-Mediated Antigen Presentation. J. Immunol..

[58] Rawson P.M., Molette C., Videtta M., Sette A., Altieri L., Franceschini D., Donato T., Paroli M., Meloni F., Sidney J. (2007). Cross-presentation of caspase-cleaved apoptotic self-antigens in HIV infection. Protoc. Exch..

[59] Sollberger G., Strittmatter G.E., Kistowska M., French L., Beer H.-D. (2012). Caspase-4 Is Required for Activation of Inflammasomes. J. Immunol..

[60] Guermonprez P., Saveanu L., Kleijmeer M.J., Davoust J., van Endert P., Amigorena S. (2003). ER–phagosome fusion defines an MHC class I cross-presentation compartment in dendritic cells. Nature.

[61] Johnsen A.K., Templeton D.J., Sy M., Harding C.V. (1999). Deficiency of transporter for antigen presentation (TAP) in tumor cells allows evasion of immune surveillance and increases tumorigenesis. J. Immunol..

[62] Chen H., Li L., Weimershaus M., Evnouchidou I., van Endert P., Bouvier M. (2016). ERAP1-ERAP2 dimers trim MHC I-bound precursor peptides; implications for understanding peptide editing. Sci. Rep..

[63] York I.A., Brehm M.A., Zendzian S., Towne C.F., Rock K.L. (2006). Endoplasmic reticulum aminopeptidase 1 (ERAP1) trims MHC class I-presented peptides in vivo and plays an im-portant role in immunodominance. Proc. Natl. Acad. Sci. USA.

