# Supplementary material for: Alterations in HLA Class I-Presented Immunopeptidome and Class I-Interactome upon Osimertinib Resistance in EGFR Mutant Lung Adenocarcinoma
Source: Cancers (Basel). 2021 Oct 4;13(19):4977. doi: 10.3390/cancers13194977 (PMC8507780; doi:10.3390/cancers13194977)

Supplementary Figure 1

a

Cell line	Database	Accession ID
H1975	ATCC	CRL-5908
PC9	MSKCC	N.A.
H1975-OsiR	In-house	N.A.
PC9-OsiR	In-house	N.A.

b

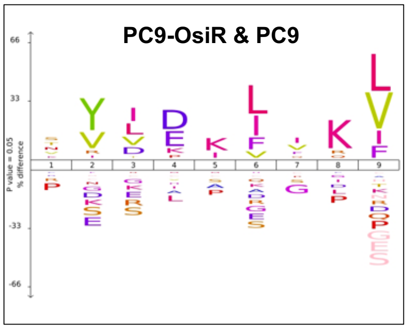

c

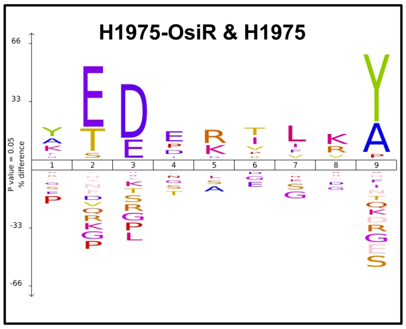

d

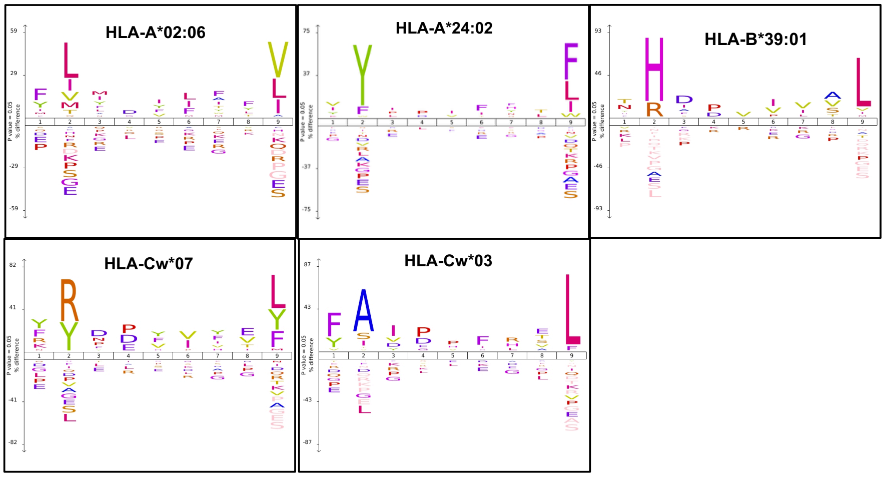

e

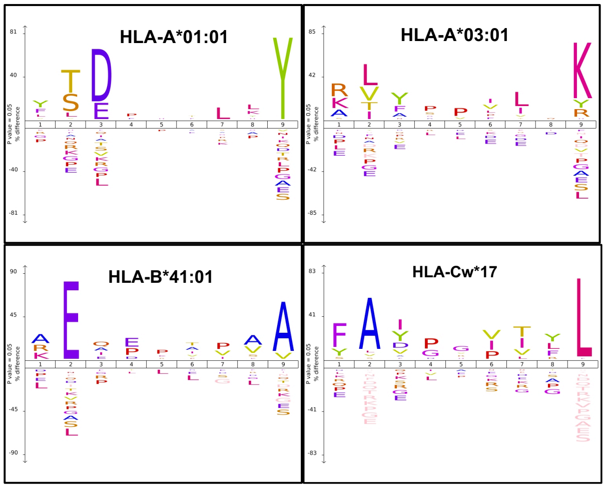

Supplement: Supplementary file 1 [file cancers-13-04977-s001.zip › Suppl Figure S1.pdf]
