# Supplementary figures and images for: Alterations in HLA Class I-Presented Immunopeptidome and Class I-Interactome upon Osimertinib Resistance in EGFR Mutant Lung Adenocarcinoma
Source: Cancers (Basel). 2021 Oct 4;13(19):4977. doi: 10.3390/cancers13194977 (PMC8507780; doi:10.3390/cancers13194977)

Supplementary Figure 3

a

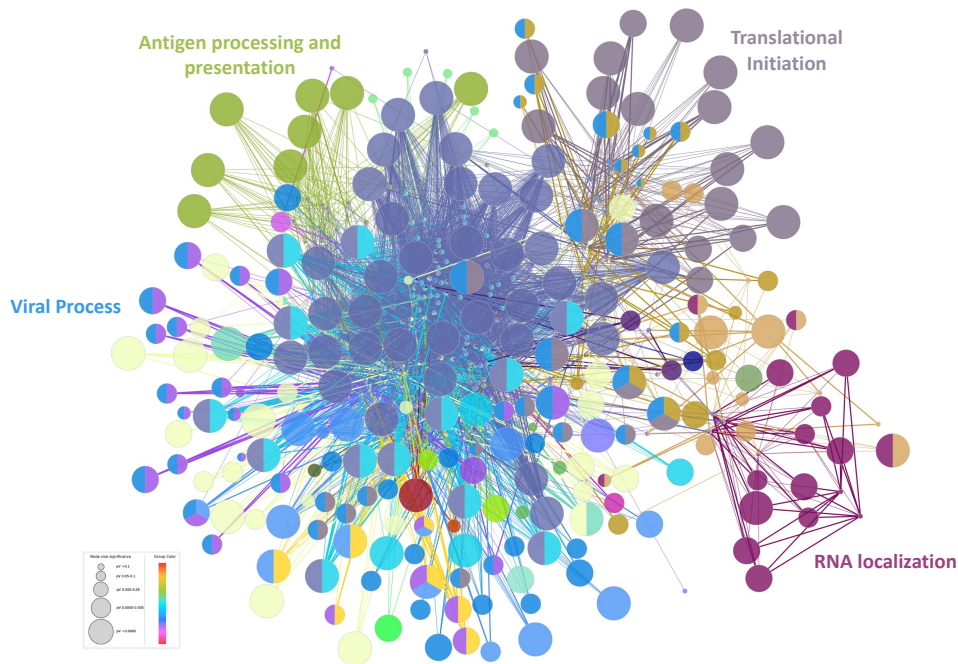

b

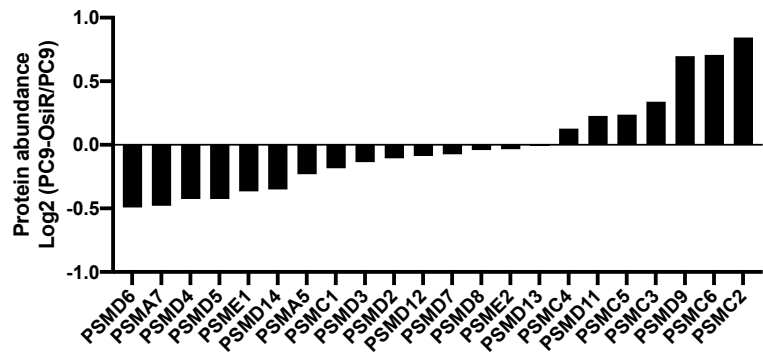

Supplement: Supplementary file 1 [file cancers-13-04977-s001.zip › Suppl Figure S3.pdf]

## Supplementary Figure 2

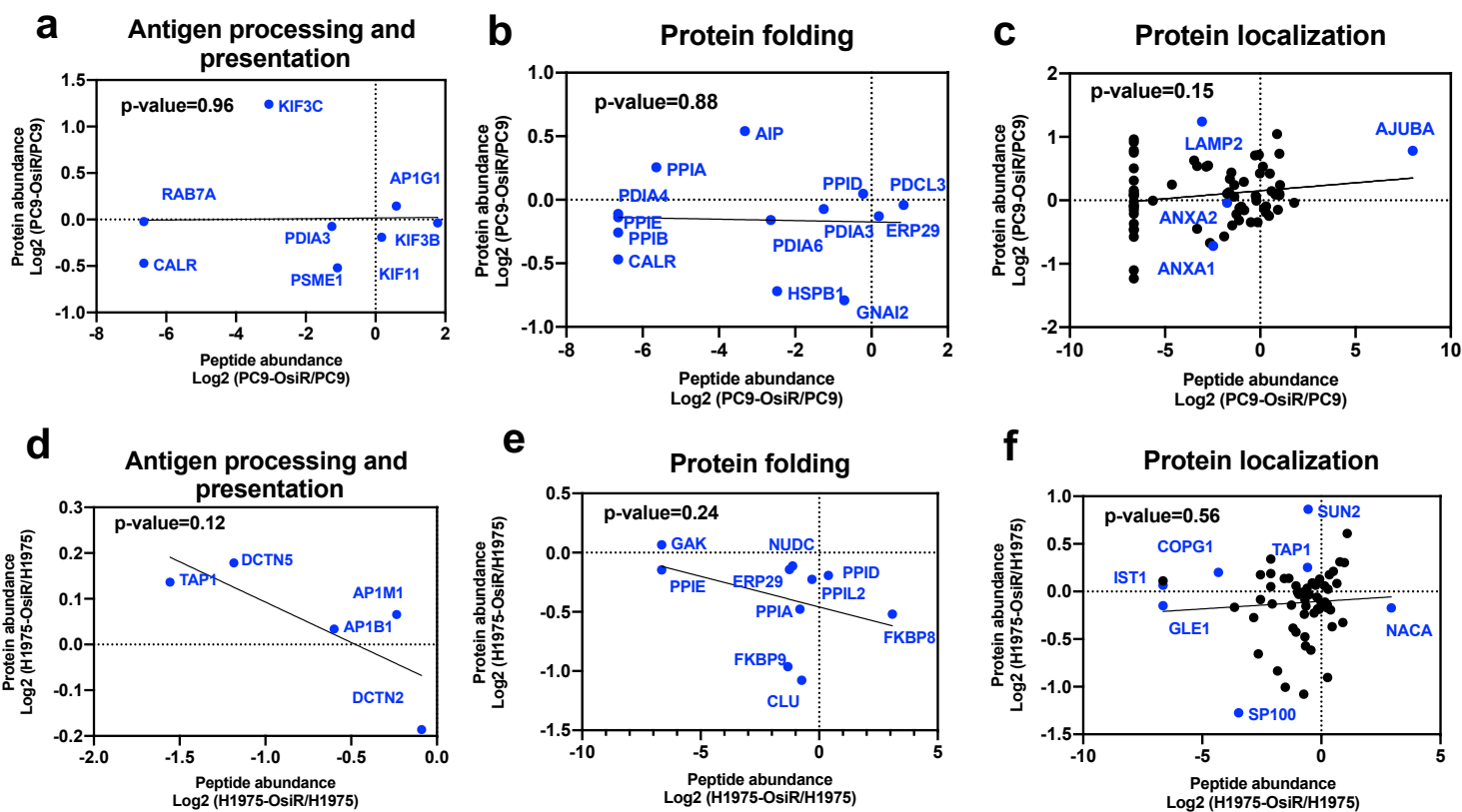

Supplement: Supplementary file 1 [file cancers-13-04977-s001.zip › Suppl Figure S2.pdf]
